# Rectal Cancer: 20% Risk Reduction Thanks to Dietary Fibre Intake. Systematic Review and Meta-Analysis

**DOI:** 10.3390/nu11071579

**Published:** 2019-07-12

**Authors:** Vincenza Gianfredi, Daniele Nucci, Tania Salvatori, Giulia Dallagiacoma, Cristina Fatigoni, Massimo Moretti, Stefano Realdon

**Affiliations:** 1 Post-Graduate School of Hygiene and Preventive Medicine, Department of Experimental Medicine, University of Perugia, P.le L. Severi 1, 06122 Perugia, Italy; 2 Digestive Endoscopy Unit, Veneto Institute of Oncology IOV-IRCCS, Via Gattamelata 64, 35128 Padua, Italy; 3 Department of Pharmaceutical Science, University of Perugia, Via del Giochetto 2, 06123 Perugia, Italy; 4 Post-Graduate School of Hygiene and Preventive Medicine, Department of Public Health, Experimental and Forensic Medicine, University of Pavia, 27100 Pavia, Italy

**Keywords:** diet, fibre, rectal cancer, dietary fibre

## Abstract

The aim of this systematic review and meta-analysis was to evaluate the association between dietary fibre intake and rectal cancer (RC) risk. In January 2019, a structured computer search on PubMed/Medline, Excerpta Medica dataBASE (EMBASE) and Scopus was performed for studies reporting the results of primary research evaluating dietary fibre intake in women and men as well as the risk of developing RC. Preferred Reporting Items for Systematic Reviews and Meta-Analyses (PRISMA) recommendations were followed. Highest vs. lowest fibre concentrations was compared. The Egger test was used to estimate publication bias. Heterogeneity between studies was evaluated with I^2^ statistics. The search strategy identified 912 papers, 22 of which were included in our meta-analysis. Having evaluated a total of 2,876,136 subjects, the results suggest a protective effect of dietary fibre intake on RC prevention. The effect Size (ES) was [0.77 (95% CI = 0.66–0.89), *p*-value = 0.001)]. Moderate statistical heterogeneity (Chi^2^ = 51.36, df = 21, I^2^ = 59.11%, *p*-value = 0.000) was found. However, no publication bias was found, as confirmed by Egger’s linear regression test (Intercept −0.21, t = −0.24, *p* = 0.816). The findings suggest that dietary fibre intake could be protective against RC, with a clinically relevant reduction of RC risk. Identifying preventive measures to avoid the development of RC, especially by following a healthy lifestyle including healthy diet, is pivotal.

## 1. Introduction

Colorectal cancer is the third most common cancer worldwide, after lung and breast cancer. It represents 10% of all cancers, excluding non-melanoma skin cancer, and it is the second leading cause of cancer-related death worldwide, accounting for approximately 1.8 million new cases and almost 900,000 deaths in 2018 [1]. Developed countries, in particular in Oceania and Europe, account for 54% of all cases. Growing evidence shows that approximately 47% of cases of colorectal cancer can be prevented by eating and drinking healthily, being physically active and maintaining a healthy weight [2]. As a matter of fact, evidence suggests that the dietary and lifestyles changes observed in recent decades are responsible for the increased incidence of chronic diseases, including cancer. As far as diet is concerned, it is already known that the Western Diet (rich in fat, sugars and animal products) is associated with an increased risk of cancer, colorectal among others [3]; on the contrary, the Mediterranean Diet (rich in fruits and vegetables, whole grains and pulses) shows a higher protective role against cancer [4]. Data from the largest European prospective cohort study (European Prospective Investigation into Cancer and Nutrition study (EPIC)) failed to support the higher protective role played by an increased intake of fruits and vegetables, compared to a healthy and balanced diet, to prevent colon cancer [5]. Nevertheless, the results from an extensive and recent meta-analysis confirm the protective role of fibre on colon cancer risk [6]. According to the World Cancer Research Fund and the American Institute for Cancer Research, a consumption of at least 90 g/day of whole grain significantly reduces the risk of colon cancer mainly due to their high fibre content [2].

Approximately one-third of cases of colorectal cancer are located in the rectum, and most of these are rectal adenocarcinomas [7]. Colorectal cancer varies a lot in terms of aetiology, prognosis and treatment, depending on its location within the colon: proximal and distal sections of the colon and rectum widely differ in their functioning mechanisms, and these differences should be taken into account when investigating the risk factors and protective strategies for colon and rectal cancer. There are several mechanisms that could be involved in the anticarcinogenic effect of dietary fibres in colorectal cancer, including physical mechanisms and prebiotics effects. Among the physical mechanisms, fibre is responsible not only for increasing the faecal bulk, thus improving the sequestration and dilution of other carcinogens to which the intestinal epithelium is exposed though diet, but it also allows for a faster intestinal transit time that results in reducing the duration of toxins exposure for the intestinal epithelium [8]. Moreover, fibres have prebiotics effects as well: prebiotics are non-digestible elements that might have beneficial effects through gut microbiota metabolization [9]. However, the studies available in literature show inconclusive results and these inconsistencies may essentially be the result of different age at diagnosis, tumour site or dose needed to observe an association [10].

Colon and rectum, in spite of their similar anatomical features and close contact, play different roles in the digestion process, and therefore have different underlying pathophysiological mechanisms. For instance, transit time is lower within the colon, compared to the rectum where the faecal mass is stored before expulsion through defecation. These differences, as well as the different homeostatic mechanisms, may play a role in the development of cancer, and should be taken into consideration when studying the colon and rectum. Nevertheless, even though several studies have investigated the association of plant food and fibre intake with colorectal cancer (involving either the whole colon and rectum or the colon alone), only a limited number of studies have examined the possible associations of these dietary habits with rectal cancer.

Although it is not easy to investigate rectal cancer alone, we performed a systematic review and meta-analysis aimed at estimating the potential association between dietary fibre intake and reduced risk of rectal cancer development.

## 2. Material and Methods

We performed this systematic review and meta-analysis following the Preferred Reporting Items for Systematic Reviews and Meta-Analyses (PRISMA) guidelines [11]. A structured computer literature search was performed on the PubMed/Medline, Excerpta Medica dataBASE (EMBASE) and Scopus databases, using a pre-determined combination of keywords, according to the type of database consulted. The selection of keywords was conducted considering three aspects: rectal cancer, fibre intake and type of study. The keywords were then combined using Medical Subject Headings (MeSH), text words, and Boolean operators AND/OR. The search terms identified were: rectal, colorectal or colon cancer and intestinal or adenomatous polyp, combined with dietary fibre, edible grain, vegetables, bread, fruit, and prospective, cross-sectional, cohort or follow-up studies, clinical trial, incidence, epidemiology surveys and questionnaires. The literature search was carried out in January 2019 and an update was conducted in May 2019.

### 2.1. Inclusion/Exclusion Criteria

The retrieved references had to report the results of primary research evaluating dietary fibre intake in women and men, as well as the risk of rectal cancer development, in order to be included in the systematic review. Furthermore, only studies published in English and for which the full text was available were included in the analysis. No time filter was added; animal model studies, non-original papers (e.g., reviews, letters to the editor, commentaries); studies reporting different outcomes and data not expressed as risks [e.g., Odds Ratio (OR), Risk Ratio (RR) or Hazard Ratio (HR)] were excluded. Moreover, because our aim was to evaluate the effect of daily dietary fibre intake, studies investigating the effect of fibre supplementations were not included in the review. Supplementary Table S1 reports the detailed inclusion/exclusion criteria according to a Population, Intervention, Comparison, Outcomes and Study design (PICOS) [12], extended with time and language filters, as recommended by the Cochrane Collaboration [13].

### 2.2. Data Extraction

All the collected studies were first screened according to the title and abstract. The full text was obtained only for potentially useful papers. Article screening and data extraction of the selected studies were conducted in double blind by two independent researchers (VG and TS). Any potential disagreement was solved through discussion between the two researchers. If the disagreement persisted, a third researcher was consulted. Data were collected only from articles satisfying the inclusion/exclusion criteria. A pre-defined spreadsheet, elaborated using Microsoft Excel^®^ for Windows, was adopted in order to systematically record the main qualitative and quantitative data extracted from the included studies. The quantitative data recorded were: amount of fibre intake, sample size, duration of the study (expressed in years), and outcome (expressed as risk). When the adjusted estimated risks were available in primary studies, these were used preferentially. Body Mass Index (BMI), smoking behaviour, gender and age were the main confounding factors considered. Qualitative data included: name of first author, year of publication, country where the study was conducted and the modality to collect dietary information. Moreover, characteristics of the subjects were also recorded (e.g., age, gender). Corresponding authors were contacted by e-mail when detailed information was not available. The reference lists were additionally screened in order to collect further related articles.

### 2.3. Quality Evaluation

Two researchers (VG and DN) independently undertook a quality evaluation of the included articles, using the checklist elaborated by The Cochrane Collaboration [13]. The Cochrane risk of bias assessment tool covers several domains of bias, including selection, detection, attrition, and performance bias. This evaluation system does not allow to calculate a numerical score, because it does not assign a point for each item. On the contrary, this tool estimates the risk of bias by assigning three different options for each item: low, high or unclear risk of bias.

### 2.4. Statistical Analysis

The effect size (ES) was calculated based on the risks (HR/OR/RR) provided per each study and it was estimated by RR (risk ratio) reported with its 95% confidence interval (CI). Because the amounts of fibre intake reported in the original studies were widely different (both in terms of units and quantities), it was not possible to compare specific quantities of fibre intake. For this reason, the comparison was performed among subjects exposed to the highest concentration of dietary fibres with those exposed to the lowest intake. Random-effect models have been applied to conduct the meta-analysis. The heterogeneity among included studies was evaluated through Chi^2^ and I^2^ tests. I^2^ values > 75% means high heterogeneity, while values between 50 and 75% are considered as moderate heterogeneity. If the I^2^ is between 25 and 50%, it is considered as low heterogeneity, and below 25% as no heterogeneity. The graphical evaluation of the Funnel plot and the Egger’s regression asymmetry test were used to estimate potential publication bias. Statistical significance was set at *p* < 0.10 [14]. If publication bias was detected, a trim and fill method was used in order to adjust for publication bias [15]. To perform the meta-analysis, the software Prometa3^®^ was used.

### 2.5. Sub-Group and Sensitivity Analysis

In order to exclude the potential overlapping effect due to the inclusion of studies referring to the same cohort of patients, a sensitivity analysis was run, excluding these data. In addition, in order to consolidate the validity of the results, a sub-group analysis was developed taking into consideration: gender (male and female separately); study design (case-control and cohort studies); duration of the study (at least 10 years) and continent where the studies were conducted (Europe, America, and Asia separately).

### 2.6. Cumulative Analysis

In order to estimate the evolution of the effect size (ES) following the publication of subsequent studies, a cumulative analysis was performed. The cumulative analysis accumulates the results from the first to the latest study, and each successive result includes a synthesis of all previous studies. This subsequent combination of the results expresses their potential consistency [16]. We performed the cumulative analysis both chronologically (adding each study according to the year of publication) and considering the sample size (from the smallest to the biggest).

### 2.7. Moderator Analysis

To explore potential moderators of these observed effects, we examined the variation in ES associated with the year of publication, categorized into three groups (the first group consisted of studies until 2004, the second between 2005 and 2010, the third from 2011 until today).

## 3. Results

### 3.1. Literature Search

**Figure 1:**
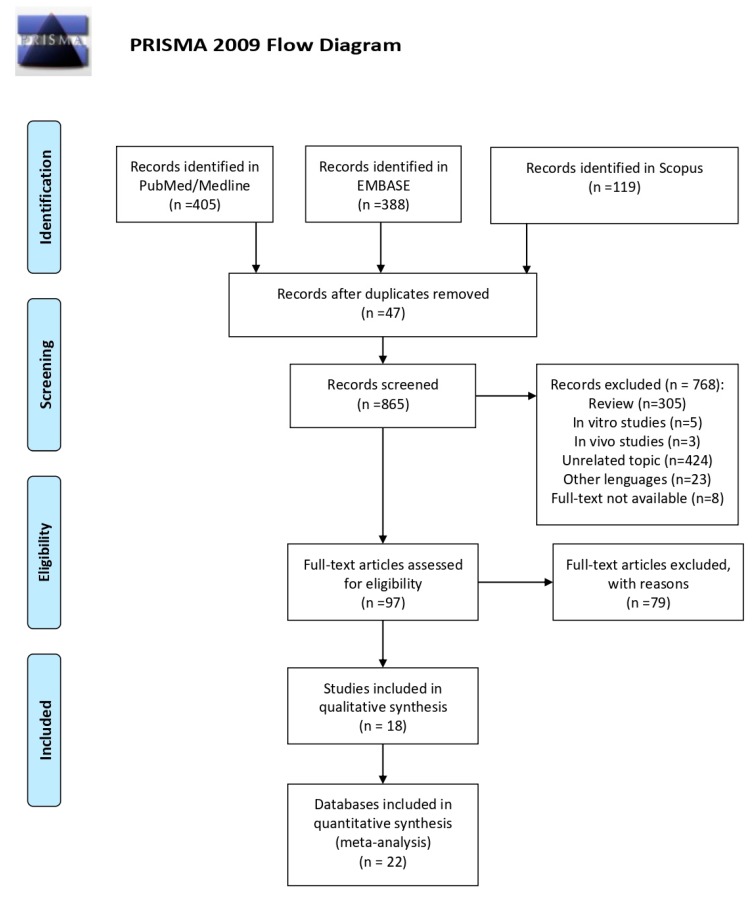
Preferred Reporting Items for Systematic Reviews and Meta-Analyses (PRISMA) flow diagram of studies selection process.

A total of 912 articles were identified, 405 of which were in the PubMed/Medline databases, 388 in the EMBASE database and 119 in the Scopus database. After the preliminary screening by title and abstract, 815 documents were excluded, because of duplicates (*n* = 47), reviews (*n* = 305), in vitro studies (*n* = 5), in vivo studies (*n* = 3), unrelated topic (*n* = 424), full-text not available (*n* = 8), different language (*n* = 23). Out of the 23 articles excluded because of language restriction, seven were written in German, five in Japanese, three in French, two in Chinese, and one in Danish, Dutch, Rumanian, Russian, Spanish and Polish. Overall, 97 articles were eligible, but 79 studies were excluded either because the selection criteria for rectal cancer were not available (data were often expressed for intestinal adenoma or for a combination of colon-rectal cancer) or because outcomes different than risk of cancer were analysed. A flow diagram, reporting the selection process, is shown in Figure 1. At the end of the screening process, 18 articles were included in the quantitative analysis; however, because four papers [10,17,18,19] reported the results divided by gender, they were considered as independent studies and, for this reason, the meta-analysis includes 22 datasets. Furthermore, because three studies reported the results both aggregated and divided by gender, we included the latter data in the sub-group analysis by gender.

### 3.2. Characteristics of Included Studies

**Figure 2:**
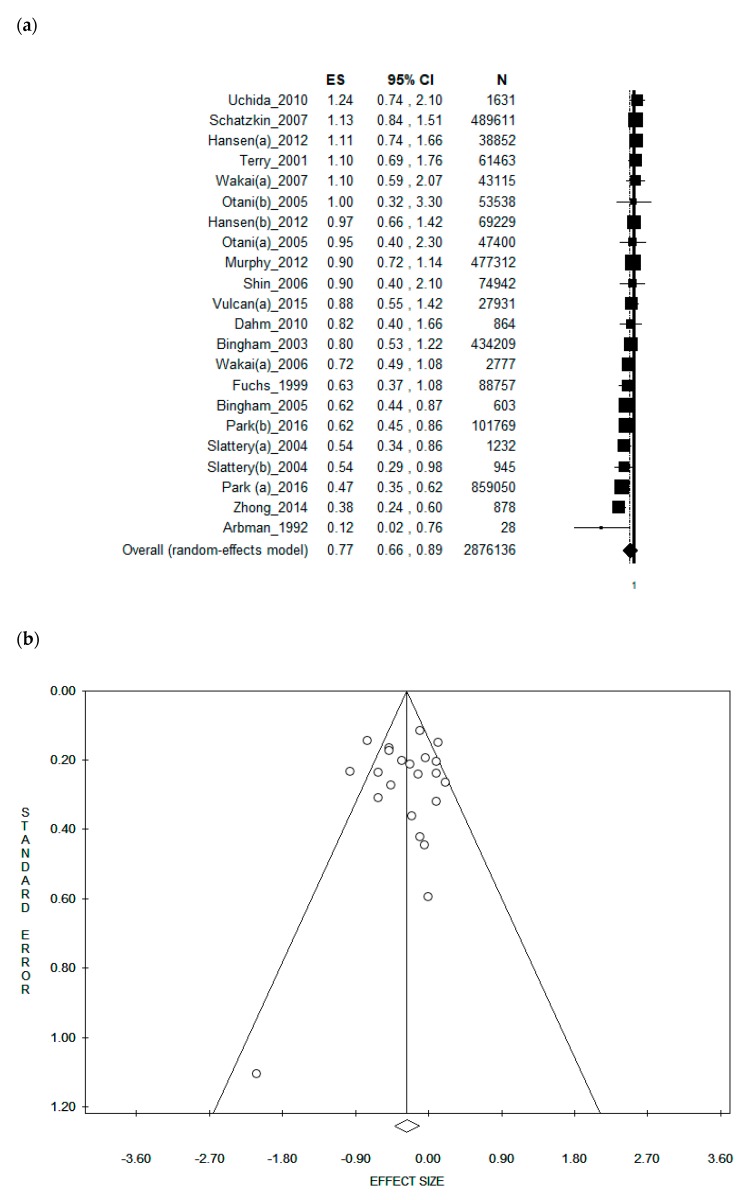
(**a**) Forest plot and (**b**) funnel plot of the meta-analysis comparing dietary fibre intake (lower vs. higher intake) in the prevention of rectal cancer.

Table 1 shows the characteristics of the studies included in the meta-analysis, alphabetically ordered and divided according to study design and gender. Supplementary Table S2 illustrates the quality evaluation. Eight studies were conducted in Europe, one of which was conducted in the United Kingdom [18,20], three in Sweden [21,22,23] and four in multi-centric European studies [19,24,25,26]. Out of these four European studies, three came from the EPIC cohort and one from the HELGA cohort [based on three prospective Scandinavian cohorts: The Norwegian Women and Cancer study (NOWAC), The Northern Sweden Health and Disease Study (NSHDS), and the Danish Diet, Cancer and Health study (DCH)]. The three EPIC cohorts included different countries: the study conducted by Bingham et al. in 2003 included France, Italy, Spain, United Kingdom, the Netherlands, Germany, Sweden and Denmark [24]; the second study, conducted by Bingham et al. in 2005, also included Norway and Sweden [25]; the study conducted by Murphy et al. in 2012 also included Greece [26]. The HELGA cohort included Norwegian, Danish and Swedish subjects [19]. In addition to these, four other studies were conducted in the United States of America (USA) [10,17,27,28], four in Japan [18,29,30,31] and two in China [32,33]. Of the included studies, five were conducted between 1992 and 2004 [10,21,22,24,27], eight were carried out in the time frame 2005–2010 [18,20,25,28,29,30,31,32], while five were published between 2011 and 2016 [17,19,23,26,33]. In 10 studies, the results were presented for males and females combined [20,21,23,24,25,28,29,30,31,33], while 10 studies reported the results for females only [10,17,18,19,22,23,27,30,31,32], and eight for males only [10,17,18,19,23,26,30,31]. The included subjects were aged between 20 and 93 years. A validated self-administered Food Frequency Questionnaire (FFQ) was used in 10 studies in order to obtain data on dietary fibre intake [18,19,22,26,27,28,30,31,33], while three studies did not specify whether the adopted questionnaire had been previously validated or not [17,24,25]; the food questionnaire was administered through an interview in two studies [21,29], while three studies used a food diary [10,20,23]. The pooled ES was [0.77 (95% CI = 0.66–0.89), *p*-value = 0.001)] based on 2,876,136 participants (Figure 2a). Moderate statistical heterogeneity (Chi^2^ = 51.36, df = 21, I^2^ = 9.11%, *p*-value = 0.000) was found. However, no publication bias was found, as demonstrated by the symmetry of the Funnel plot and confirmed by Egger’s linear regression test (Intercept −0.21, t = −0.24, *p* = 0.816) (Figure 2b).

### 3.3. Sensitivity Analysis

In order to estimate the effect of fibre intake without potential overlapping cohort studies, two of the three multi-centric EPIC cohort studies were excluded. Bingham et al. 2003 and Bingham et al. 2005 were excluded, while Murphy et al. 2012 was included because it was the most recent EPIC study and the number of included countries was higher compared to the previous ones [24,25,26]. Moreover, because Dahm et al. 2010 used national data from the EPIC cohort, this study was excluded as well [20]. However, no significant differences were found. The pooled ES was [0.78 (95% CI = 0.65–0.93), *p*-value = 0.005)] based on 2,440,460 participants, and moderate statistical heterogeneity (Chi^2^ = 49,70, df = 18, I^2^ = 63.78%, *p*-value = 0.000) was found. No publication bias was found as confirmed by Egger’s linear regression test (Intercept −0.31, t = −0.32, *p* = 0.753).

### 3.4. Sub-Group Analysis by Gender

**Figure 3:**
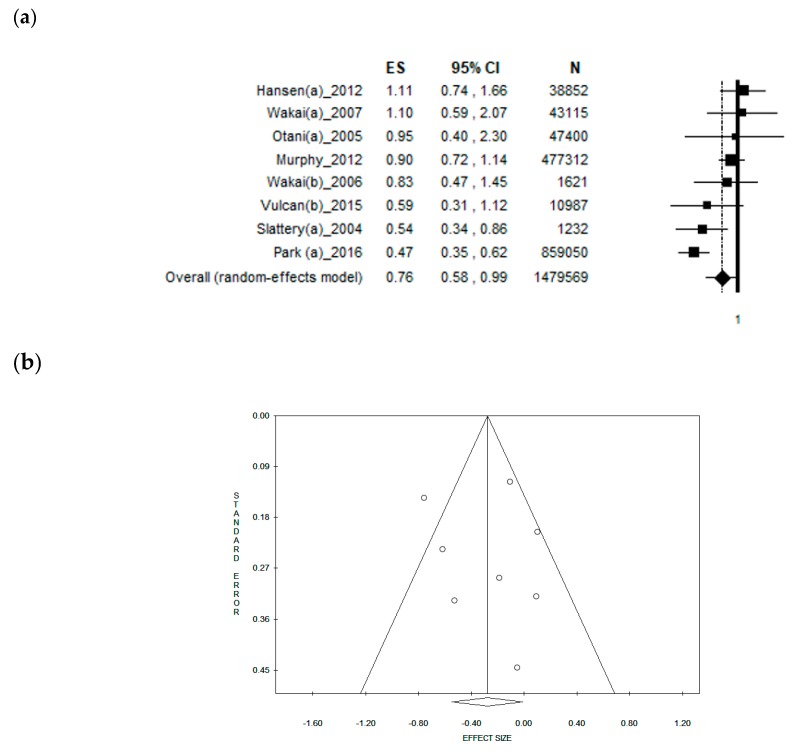
(**a**) Forest plot and (**b**) funnel plot of the meta-analysis comparing dietary fibre intake in the prevention of rectal cancer in men.

The analysis including only studies that reported separate data for males obtained a pooled ES = [0.76 (95% CI = 0.58–0.99), *p*-value = 0.042)] based on 1,479,569 men, with a moderate statistical heterogeneity (Chi^2^ = 20.59, df = 7, I^2^ = 66.00%, *p*-value = 0.004 (Figure 3a). However, the symmetry of the funnel plot shows no potential publication bias, confirmed by Egger’s linear regression test (Intercept −0.36, t = 0.22, *p* = 0.830) (Figure 3b). When analysing only the studies reporting data selectively for females, a borderline, weak, inverse association was found with an ES = [0.83 (95% CI = 0.67–1.02), *p*-value = 0.078)] based on 495,222 women. However, no statistical heterogeneity was found (Chi^2^ = 11.87, df = 9, I^2^ = 24.17%, *p*-value = 0.221). The funnel plot was symmetrical as confirmed by Egger’s linear regression test (Intercept 1.24, t = 1.31, *p* = 0.226).

### 3.5. Sub-Group Analysis by Study Type

**Figure 4:**
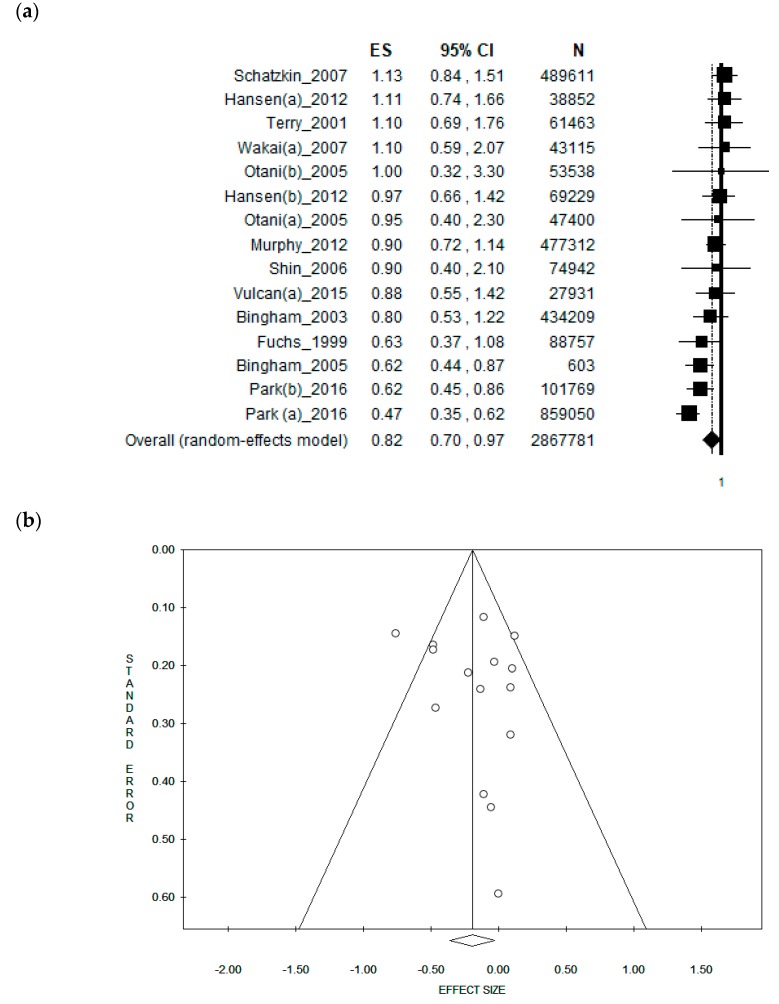

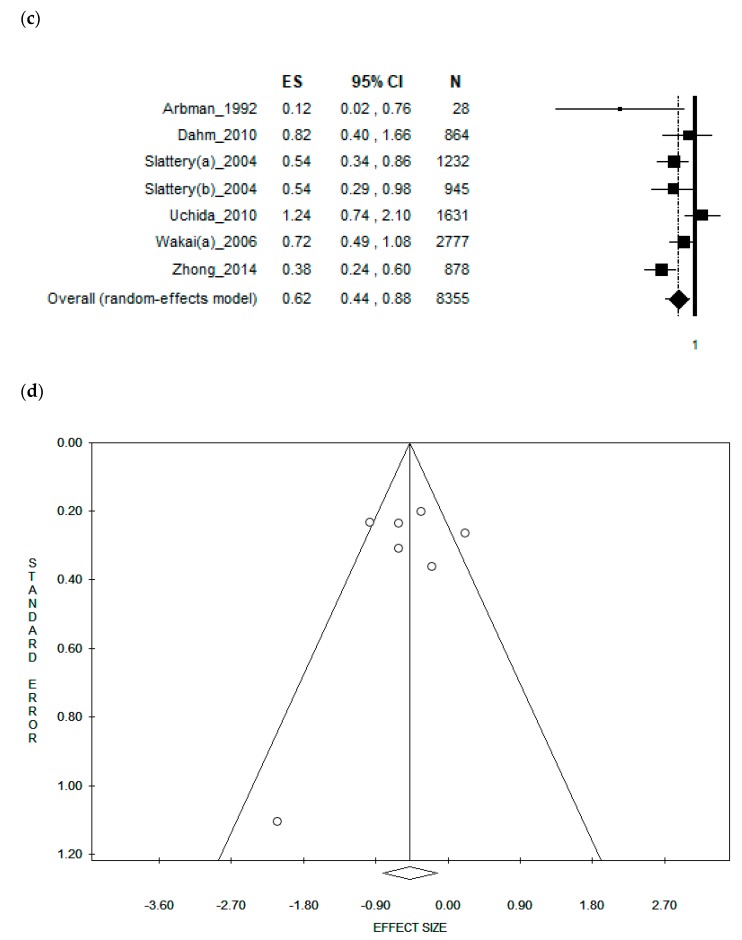
(**a**) Forest plot and (**b**) funnel plot of the meta-analysis comparing dietary fibre intake in the prevention of rectal cancer considering cohort studies. (**c**) Forest plot and (**d**) funnel plot of the meta-analysis comparing dietary fibre intake in the prevention of rectal cancer considering case-control studies.

Taking into consideration only cohort studies, the pooled ES was [0.82 (95% CI = 0.70–0.97), *p*-value = 0.021)] based on 2,867,781 individuals (Figure 4a). Moderate statistical heterogeneity (Chi^2^ = 31.68, df = 14, I^2^ = 55.81%, *p*-value = 0.004) was found. The Funnel plot was symmetrical, showing no potential publication bias, as confirmed by Egger’s linear regression test (Intercept 0.74, t = 0.69, *p* = 0.501) (Figure 4b).

Furthermore, because of the potential overlapping effect, we removed studies using the same cohort. A borderline, weak inverse association was found with an ES = [0.85 (95% CI = 0.71–1.03), *p*-value = 0.093)] based on 2,432,969 participants. Moderate statistical heterogeneity (Chi^2^ = 29.14, df = 12, I^2^ = 58.82%, *p*-value = 0.004) was found. No publication bias was found, as confirmed by Egger’s linear regression test (Intercept 0.71, t = 0.63, *p* = 0.542).

When only case-control studies were included in the analysis, the pooled ES was [0.62 (95% CI = 0.44–0.88), *p*-value = 0.007)] based on 8355 participants (Figure 4c). Moderate statistical heterogeneity (Chi^2^ = 15.04, df = 6, I^2^ = 60.10%, *p*-value = 0.020) was found. No publication bias was found as confirmed by the symmetry of the Funnel plot and by the Egger’s linear regression test (Intercept −1.06, t = −0.57, *p* = 0.596) (Figure 4d).

### 3.6. Sub-Group Analysis by Duration of the Studies

**Figure 5:**
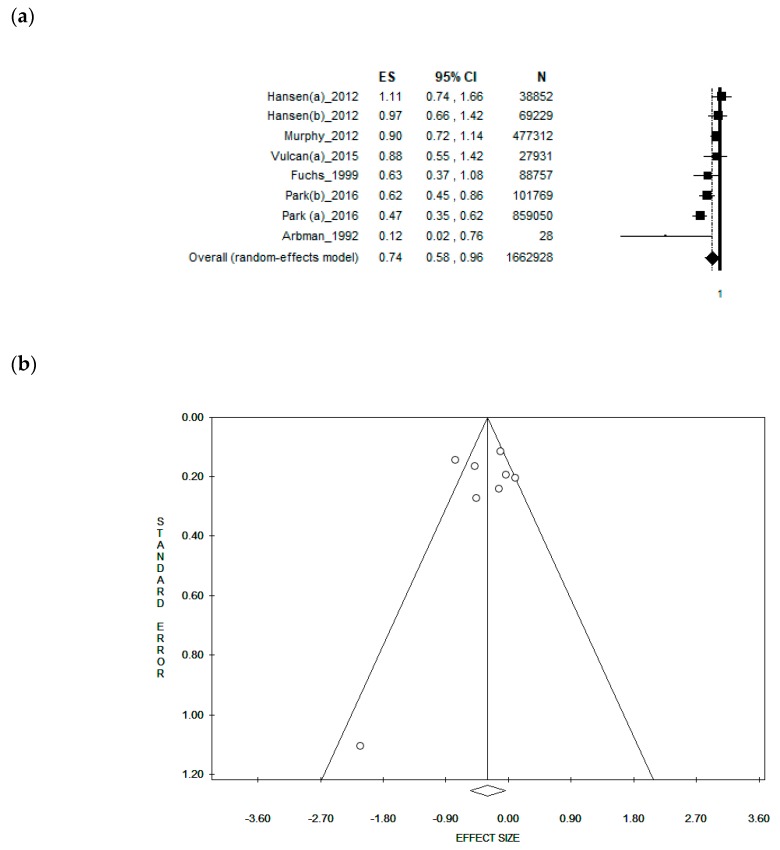
(**a**) Forest plot and (**b**) funnel plot of the meta-analysis comparing dietary fibre intake in the prevention of rectal cancer considering only studies with at least 10 years of observation.

Including only studies with at least 10 years of observation, the pooled ES was [0.75 (95% CI = 0.59–0.95), *p*-value = 0.017)] based on 1,663,792 participants (Figure 5a). Moderate statistical heterogeneity (Chi^2^ = 23.01, df = 8, I^2^ = 65.23%, *p*-value = 0.003) was found. However, no publication bias was found as shown in the Funnel plot and confirmed by Egger’s linear regression test (Intercept −0.63, t = −0.43, *p* = 0.679) (Figure 5b).

### 3.7. Sub-Group Analysis by Continent

**Figure 6:**
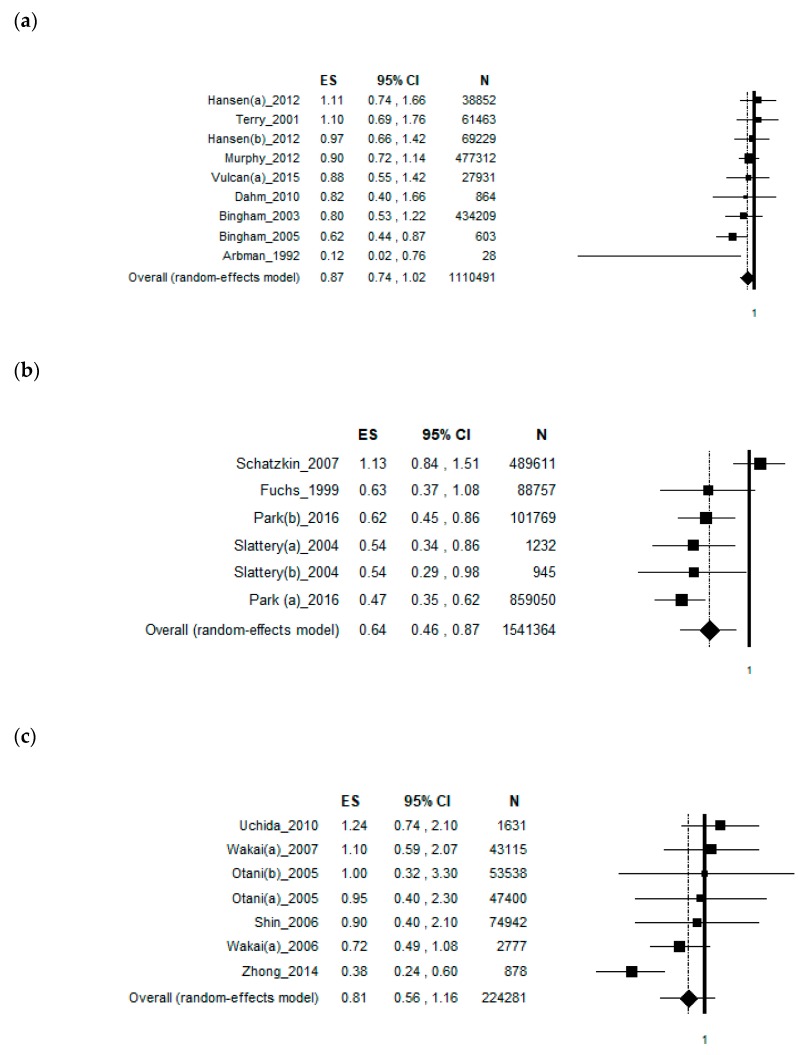
(**a**) Forest plot of the meta-analysis considering only studies conducted in Europe, (**b**) forest plot of the meta-analysis considering only studies conducted in America and (**c**) forest plot of the meta-analysis considering only studies conducted in Asia.

The included studies were conducted in Europe, America and Asia. The analysis including only studies conducted in Europe obtained a borderline, weak, inverse association with an ES = [0.87 (95% CI = 0.74–1.02), *p*-value = 0.079)] based on 1,110,491 subjects, with no statistical heterogeneity (Chi^2^ = 9.94, df = 8, I^2^ = 19.54%, *p*-value = 0.269) (Figure 6a). The symmetry of the Funnel plot shows no potential publication bias, confirmed by Egger’s linear regression test (Intercept -0.91, t = −0.97, *p* = 0.367). When analysing only the studies conducted in America, a significant inverse association was found with an ES = [0.64 (95% CI = 0.46–0.87), *p*-value = 0.005)] based on 1,541,364 subjects. A moderate statistical heterogeneity was found (Chi^2^ = 19.64, df = 5, I^2^ = 74.54%, *p*-value = 0.001) (Figure 6b). The Funnel plot was symmetrical as confirmed by Egger’s linear regression test (Intercept −1.50, t = −0.47, *p* = 0.663). Considering only the studies conducted in Asia, a borderline, weak, inverse association with an ES = [0.95 (95% CI = 0.81–1.11), *p*-value = 0.247)] based on 224,281 subjects, with moderate statistical heterogeneity (Chi^2^ = 14.20, df = 6, I^2^ = 57.75%, *p*-value = 0.027) was found (Figure 6c). The symmetry of the Funnel plot shows no potential publication bias, confirmed by Egger’s linear regression test (Intercept 1.76, t = 0.98, *p* = 0.371).

### 3.8. Cumulative and Moderator Analysis

The cumulative analysis, shown in Supplementary Figure S1a, revels that early studies, even if suggesting an inverse association between fibre intake and risk of rectal cancer, had a large 95% CI, without statistical significance. However, in the early 2000s, the results started to become stable, statistically significant and with a narrow 95% CI. Referring to the cumulative analysis for sample size (Supplementary Figure S1b), small studies with a sample size of less than 2000 subjects did not find any statistically significant association. A higher number of participants contributed to stabilize the results thought a reduction of the 95% CI and a confirmation of the ES value.

Considering the year of publication as covariate in the moderator analysis, the results did not change significantly between the three groups of years, as shown in Supplementary Figure S2.

## 4. Discussion

This paper reports the results of an extensive systematic review and meta-analysis conducted using three scientific databases (PubMed/Medline, EMBASE and Scopus). Out of 912 retrieved studies, 18 papers were included in the quantitative and qualitative evaluation; however, because some of them reported separate data for males and females, the whole sample was based on 22 databases. The original papers included in the analysis were mainly studies conducted in western countries, while six were performed in Asia (the subgroup analysis by continent shows a higher inverse association in studies conducted in western countries compared to the Asiatic studies). The findings from this systematic review and meta-analysis suggest that dietary fibre intake could be protective against rectal cancer, with a clinically relevant reduction of rectal cancer risk [ES = 0.77 (95% CI = 0.66–0.89)]. The consistency of this result has been confirmed after removing studies using same cohort population. The subgroup analysis by gender shows a higher protective effect for males, compared to females, although this could be due to the different sample size between the two groups: in fact, the analysis conducted among males involved a total of 1,479,569 men, compared to 495,222 females. Furthermore, the highest strength of the association between dietary fibre intake and rectal cancer was found focusing on case-control studies. This could be due to the natural history of rectal cancer, characterized by a long latency, which is better appraised using a case-control study design. Nevertheless, case-control studies are more prone to potential recall bias; however, the high number of participants included and the time frame of observation might mitigate this disadvantage. On the contrary, cohort studies are more prone to selection bias, and maintaining a follow up might be difficult, especially in studies where the outcome requires a long duration of the follow-up [34]. Because of the above-mentioned aspects, studies included in this meta-analysis reported mixed findings between dietary fibre intake and rectal cancer risk. Nevertheless, not all the studies included had a large sample size and long follow-up time to examine dietary associations: as a matter of fact, when we performed the sub-group analysis, restricted to studies with at least 10 years of observation, the risk reduction was higher.

Even though dietary fibre is resistant to human enzyme digestion, it does not pass through the intestinal tract without any effects. The protective effect of dietary fibre and the risk of developing rectal cancer have to be ascribed both to physical mechanisms and prebiotics effects. The gut microbiota is able to metabolize some toxic food compounds (for instance heterocyclic amines), which can be directly bound or transformed into less noxious elements, thus reducing the cancerogenic risk for the host’s intestinal epithelium. In addition to this, flourishing evidence shown a relationship between dietary fibre structure and fermentation rate. It seems that the fermentation rate is modified by the type and mixture of dietary fibres ingested. In particular, fast-fermenting fibres, (i.e., fibres from citrus) delay the fermentation of other gut bacteria [35], due to a hierarchical preference in gut bacteria’s fermentation ability [36]. This hierarchical preference affects the fermentation rate, that helps the fibres to reach the distal part of the intestinal tract, allowing them to perform their biological effect in the rectum [37]. This aspect is particularly important in terms of rectal cancer risk. Moreover, stimulating the formation of short-chain fatty acids (SCFA), such as butyrate and acetate, from fermentation by intestinal bacteria, might stimulate the hepatic detoxification process [8]. Moreover, because dietary fibres mainly came from fruits, vegetables and pulses, they might release bioactive compounds such as phenols [38]. Some evidence also suggests a potential negative effect played by gut microbiota. This happens mainly in case of dysbiosis (alteration of microbiota), during which pathogenic microorganisms are isolated [39]. Dysbiosis has been associated with several diseases, including colorectal cancer [40]. Since colon and rectal cancers are mostly addressed together as colorectal cancer, only a limited amount of data related to dysbiosis in colon and rectal cancers separately is available.

However, recent studies showed a different microbiota impairment in colon or rectal cancer. Indeed, a reduction of Lactobacillaceae was related to colon cancer, while a reduction in Bifidobacteriaceae abundance and a significant increase of the genus *Alistipes, Akkermansia, Halomonas* and *Shewanella* were related to rectal cancer [41,42]. The role of Bifidobacteriaceae in protecting and maintain gut microbial environment is well established [43]. Thus, a loss in Bifidobacterium abundance seems to result in a decreased competition against pathogens, impaired immune modulation function, impaired fibre fermentation with loss in production of acetate and a decreased synthesis of folate that might contribute to chromosomal instability [43,44].

Therefore, intestinal health is based on the balance between host’s diet and pool of commensal microorganisms. The microbiota profile varies largely between individuals and, even though the healthy microbiota has not been completely defined yet, evidence has confirmed the impact of diet on this [45]. Albeit, the above-mentioned considerations are applicable for both colon and rectum, which have different underlying physiological functions. The rectum is much more exposed to genotoxic and cytotoxic damages due to the longer transit time compared to the colon, and to the faecal mass storage before expulsion through defecation. Moreover, the water content (and therefore the toxins/nutrients concentrations) varies as well, the rectal bulk being more compact and less hydrated.

### Strengths and Limitations of the Study

Before generalizing the results of this meta-analysis, some aspects need to be taken into consideration. Dietary fibre intake was mainly assessed through questionnaires, and even if FFQ is a consolidated method, not all the included studies used a validated questionnaire. Moreover, using an FFQ, recall bias cannot be excluded, while the food diary and the 24-hour recall are gold-standard tools. However, because the food intake is mainly self-reported, regardless of the instrument used, social desirability bias cannot be eliminated. Because of recall and social desirability bias, misclassification or under/over-reporting of fibre intake might result in a modified grade of association. Furthermore, no information on processing methods are reported in the original studies, and according to recent evidence, the processing method is associated with alteration of fibre microstructure that in turn is associated with changes in biological effects [46,47]. All the above-mentioned limits are mainly due the primary study design, and even if we conducted a risk of bias evaluation and several adaptations (for instance, groups analysis and adjusted risk), it is not possible to estimate the impact of this potential misclassification. Furthermore, in the moderator analysis, it was not possible to choose other covariates, such as age or the amount of fibre intake, because they were not uniform through the original studies. Another possible limitation of this meta-analysis is the fact that only studies published in English were included, even though this is the universal language of the scientific community and the most relevant articles are published in English. However, the absence of publication bias found, leads us to think that no relevant studies are missed. In spite of this, this review and meta-analysis has some strengths, starting from the large number of participants (2,876,136), including both women and men, and the wide range of age (20–93 years old). Moreover, in our meta-analysis, the rectal cancer estimated risks were adjusted for confounders such as BMI, smoking behaviour, gender, age, and continent where the studies were performed. Lastly, the subgroup analysis was conducted not only for study design, but also for duration of the study and gender. As a matter of fact, the knowledge on intervention effectiveness in different subgroups is particularly important for decision-makers in Public Health. Particularly, men have a higher risk of colorectal cancer compared to women, and at the same time–in this meta-analysis–men represent the group for which fibre intake plays a significantly protective effect. However, due to the low number of women included in this type of evaluation, further epidemiological studies are needed to corroborate these results. To the best of our knowledge, this is the first systematic review and meta-analysis selectively assessing the association between dietary fibre intake and risk of rectal cancer. Moreover, we estimated the effect separately for both sexes. In addition, the use of a pre-defined data extraction form and the extraction process performed independently by two reviewers, not only increased the validity and reliability of this study, but also reduced the risk of bias. Lastly, because of the low/moderate level of heterogeneity and the absence of publication bias, we can consider the systematic review and meta-analysis widely exhaustive.

## 5. Conclusions

In conclusion, our findings confirm the potential beneficial association between dietary fibre consumption and a reduced risk of colon cancer, particularly in men. Nutritional educational programmes should be improved in order to educate people on the major impact of diet on health and on the potential beneficial effects of having a healthy diet.

## Figures and Tables

**Table 1:** Characteristics of the included studies divided by study design.

Author and Year	Country	Sample Size	Gender and Age	Population Characteristics	Duration of Study	Dietary Fibre Intake * ± SD	Tool	Effect Size	*p*-Value
**Case-control studies**									
Arbman, 1992	Sweden	28	M,F 49–77 y	Patients surgically treated for rectal cancer	15 y	Cases: 21.23 ± 1.26 g/10 MJ (SEM)	dietary history protocol (interview)	RR: 0.12 (90% CI 0.02–0.76)	n.s.
						Control: 21.18 ± 1.15 g/10 MJ (SEM)			
Dahm, 2010	UK	864	M,F mean 56.97 y	Seven established UK cohorts: EPIC-Norfolk, EPIC-Oxford, the Guernsey Study, MRCNSHD, the OVS, the UKWCS and Whitehall II	21 y	Cases: 8.9 ± 1.6 g/MJ	validated Food diary	OR (95%CI) 0.82(0.40–1.66)	*p* = 0.7
						Control: 24.1 ± 5.4 g/MJ			
Slattery (a), 2004	USA	1232	M 30–79 y	KPMCP	4 y	Cases: ≤16 g/d	CARDIA diet history	OR (95%CI): 0.54(0.34–0.86)	*p* < 0.01
						Control: >34 g/d			
Slattery (b), 2004	USA	945	F 30–79 y	KPMCP	4 y	Cases: ≤16 g/d	CARDIA diet history	OR (95%CI): 0.54(0.29–0.98)	*p* < 0.01
						Control: >34 g/d			
Uchida, 2010	Japan	1631	M, F 20–74 y	Fukuka Colorectal Cancer Study, patients with histologically confirmed incident adenocarcinomas	3 y	Cases: 9.2 g/d	validated computer-assisted interview	OR (95%CI): 1.24(0.74–2.10)	*p* = 0.41
						Control: 19.8 g/d			
Wakai (a), 2006	Japan	2777	M, F 20–79 y	HERPACC included patients with cancer of colon or rectum and controls free of cancer	3 y	n.a.	validated FFQ	OR (95%CI): 0.72 (0.49–1.08)	*p* = 0.64
Wakai (b), 2006	Japan	1621	M 20–79 y	HERPACC included patients with cancer of colon or rectum and controls free of cancer	3 y	n.a.	validated FFQ	OR (95%CI): 0.83(0.47–1.45)	*p* = 0.68
Wakai (c), 2006	Japan	1156	F 20–79 y	HERPACC included patients with cancer of colon or rectum and controls free of cancer	3 y	n.a.	validated FFQ	OR (95%CI): 0.78(0.40–1.51)	*p* = 0.64
Zhong, 2014	China	878	M, F mean age cases: 56.7 ± 10.6 (SD); age controls: 56.4 ±10.5 y	Colorectal cancer patients diagnosed no more than 3 months before interview.	2 y	M/F cases: 7.13/13.79 g/d	validated FFQ	OR (95%CI): 0.38 (0.24–0.60)	*p* < 0.01
						M/F control: 14.92 /12.65 g/d			
**Cohort studies**									
Bingham, 2003	Europe	434,209	M,F 25–70 y	EPIC	6 y	Quintile 1: 12.71 g/d Quintile 5: 33.76 g/d	country-specific dietary questionnaire	HR (95% CI): 0.80 (0.53–1.22)	*p* = 0.319
Bingham, 2005	Europe	603	M, F 25–70 y	EPIC	6.2 y	Quintile 1: 17.1 g/d Quintile 5: 27.2 g/d	self-administered dietary questionnaire	HR (95% CI): 0.62 (0.44–0.87)	*p* = 0.01
Fuchs, 1999	USA	88,757	F 34–59 y	Women, participants of the Nurses’ Health Study without history of cancer, inflammatory bowel disease, or familial polyposis	16 y	Quintile 1: 9,8 ± 1,7 g/d	validated FFQ	RR (95%CI): 0.63(0.37–1.08)	*p* = 0.37
						Quintile 5: 24.9 ± 5.5 g/d			
Hansen (a), 2012	Scandinavia	38,852	M 30–64 y	HELGA	11.3 y	Quartile 1: >16.8–≤22.1 g/d	validated FFQ	RR (95%CI): 1.11(0.74–1.66)	n.s.
						Quartile 4: >28.1 g/d			
Hansen (b), 2012	Scandinavia	69,229	F 30–64 y	HELGA	11.3 y	Quartile 1: >15.4–≤19.6 g/d	validated FFQ	RR (95%CI): 0.97(0.66–1.42)	n.s.
						Quartile 4: >24.5 g/d			
Murphy, 2012	Europe	477,312	M, ≥35 y	EPIC	mean follow-up: 11.0 y	Quintile 1: <16.4 g/d	validated FFQ	HR (95%CI): 0.90(0.72–1.14)	*p* = 0.34
						Quintile 5: ≥28.5 g/d			
Otani (a), 2005	Japan	47,400	M 40–69 y	Cohort I of JPHC study started in 1990, the Cohort II started in 1993	5.8 y follow-up	Quintile 1: 6.4 g/d	validated FFQ self-administered	HR (95%CI): Highest 0.95(0.40–2.3)	*p* = 0.99
						Quintile 5: 18.7 g/d			
Otani (b), 2005	Japan	53,538	F 40–69 y	Cohort I of JPHC study started in 1990, the Cohort II started in 1993	5.8 y follow-up	Quintile 1: 8.3 g/d Quintile 5: 20.0 g/d	validated FFQ self-administered	HR (95%CI): Highest 1.0(0.32–3.3)	*p* = 0.82
Park 2016 (a)	USA	85,905	M 45–75 y	African American, Native Hawaiian, Japanese American, Latino, and white	19 y	Quintile 1: <8.1 g/1000 kcal	self-administered quantitative FFQ	HR (95%CI): Highest 0.47 (0.35–0.62)	*p* < 0.0001
						Quintile 5: >15.3 g/1000 kcal			
Park 2016 (b)	USA	101,769	F 45–75	African American, Native Hawaiian, Japanese American, Latino, and white cancer registries	19 y	Quintile 1: <8.1 g/1000 kcal	self-administered mailed questionnaire	HR (95%CI): Highest 0.62 (0.45–0.86)	*p* = 0.004
						Quintile 5: >15.3 g/1000 kcal			
Schatzkin, 2007	USA	489,611	M,F 50–71 years	NIH-AARP Diet and Health Study	5 y follow-up	Quintile 1: 6.6 g/1000 kcal	validated FFQ self-administered	RR (95%CI): 1.13(0.84–1.51)	*p* = 0.39
						Quintile 5: 15.9 g/1000 kcal			
Shin, 2006	China	74,942	F 40–72 years	SWHS	3 y	Quintile 1: 7.3 g/d Quintile 5: >13.45 g/d	validated FFQ	RR (95%CI): 0.9(0.4–2.1)	*p* = 0.335
Terry, 2001	Sweden	61,463	F 40–74 years	SMSC	9.6 y follow-up	Quartile 1: 5.7 ± 1.4 g/d	validate FFQ (self-administered)	RR (95%CI): 1.10(0.69–1.76)	*p* = 0.15
						Quartile 4: 13.6 ± 2.7 g/d			
Vulcan (a), 2015	Sweden	27,931	M, F 66-93 years	MDCS	15.4 follow-up y	Quintile 1: 0–1.7 g/MJ	7-d menu book	HR (95% CI): 0.88(0.55–1.42)	*p* = 0.525
						Quintile 5: 2.7–8 g/MJ			
Vulcan (b), 2015	Sweden	10,987	M 66–93 years	MDCS	15.4 follow-up y	Quintile 1: 0–1.7 g/MJ	7-d menu book	HR (95% CI): 0.59(0.31–1.12)	*p* = 0.263
						Quintile 5: 2.7–8 g/MJ			
Vulcan (c), 2015	Sweden	16,944	F 66–93 years	MDCS	15.4 follow-up y	Quintile 1: 0–1.7 g/MJ	7-d menu book	HR (95% CI): 1.36(0.67–2.78)	*p* = 0.660
						Quintile 5: 2.7–8 g/MJ			
Wakai (a), 2007	Japan	43,115	M,F 40–79 y	JACC	7.6 y	Quartile 1: 7.1 ±2.0 g/d	validated FFQ	RR (95%CI): 1.10 (0.59–2.07)	*p* = 0.67
						Quartile 4: 13.4 ± 2.9 g/d			
Wakai (b), 2007	Japan	16,636	M, 40–79 y	JACC	7.6 y	Quartile 1: 6.7 ± 2.0 g/d	validated FFQ	RR (95%CI): 0.95 (0.45–2.02)	*p* = 0.89
						Quartile 4: 13.4 ± 3.0 g/d			
Wakai (c), 2007	Japan	26,479	F, 40–79 y	JACC	7.6 y	Quartile 1: 7.4 ± 2.1 g/d	validated FFQ	RR (95%CI): 1.82 (0.59–5.65)	*p* = 0.19
						Quartile 4: 13.4 ± 2.8 g/d			

Abbreviations: F = female, M = male, Y = years, HR = Hazard Ratio, OR = Odd Ratio, RR = Relative Risk, SD = Standard Deviation, SEM = Standard error of the mean, KPMCP = Kaiser Permanente Medical Care Program of Northern California, OVS = Oxford Vegetarian Study, UKWCS = UK Women’s Cohort Study, EPIC = European Prospective Investigation into Cancer and Nutrition study, JACC = Japan Collaborative Cohort Study, MDCS = Malmo Diet and Cancer Study, population-based prospective study, SMSC = Swedish Mammography Screening Cohort, SWHS = Shanghai Women’s Health Study, JPHC = Japan Public Health Center-Based Prospective Study, NOWAC = Norwegian Women and Cancer study, NSHDS = The Northern Sweden Health and Disease Study, DCH = Danish Diet, Cancer and Healthy Study, HELGA = consists of people from NOWAC, NSHDS, and DCH cohorts: MRCNSHD = Medical Research Council National Survey of Health and Development, * Dietary fibre intake has been reported as a mean or range, based on the values expressed in the original paper.

## Supplementary Materials

The following are available online at https://www.mdpi.com/2072-6643/11/7/1579/s1, Table S1: Detailed inclusion/exclusion criteria, according to PICOS statement extended with language and time filter; Table S2: Quality evaluation of included studies (Low, Unclear, High); Figure S1a: Forest plot of the cumulative analysis for year of publication; Figure S1b: Forest plot of the cumulative analysis for sample size in prevention of rectal cancer; Figure S2: Forest plot of the moderator analysis for year of publication in prevention of rectal cancer.

## References

[1] Internation Agency for Reseach on Cancer Colorectum: Factsheet. https://gco.iarc.fr/today/data/factsheets/populations/900-world-fact-sheets.pdf.

[2] World Cancer Research Fund International/American Institute for Cancer Research (2017). Countinous Update Project Report: Diet, Nutrition, Physical Activity and Colorectal Cancer.

[3] Moss A., Nalankilli K. (2017). The association between diet and colorectal cancer risk: Moving beyond generalizations. Gastroenterology.

[4] Farinetti A., Zurlo V., Manenti A., Coppi F., Mattioli A.V. (2017). Mediterranean diet and colorectal cancer: A systematic review. Nutrition.

[5] Zamora-Ros R., Barupal D.K., Rothwell J.A., Jenab M., Fedirko V., Romieu I., Aleksandrova K., Overvad K., Kyro C., Tjonneland A. (2017). Dietary flavonoid intake and colorectal cancer risk in the European prospective investigation into cancer and nutrition (EPIC) cohort. Int. J. Cancer.

[6] Gianfredi V., Salvatori T., Villarini M., Moretti M., Nucci D., Realdon S. (2018). Is dietary fibre truly protective against colon cancer? A systematic review and meta-analysis. Int. J. Food Sci. Nutr..

[7] Gaertner W.B., Kwaan M.R., Madoff R.D., Melton G.B. (2015). Rectal cancer: An evidence-based update for primary care providers. World J. Gastroenterol..

[8] Nogacka A.M., Gomez-Martin M., Suarez A., Gonzalez-Bernardo O., de Los Reyes-Gavilan C.G., Gonzalez S. (2019). Xenobiotics formed during food processing: Their relation with the intestinal microbiota and colorectal cancer. Int. J. Mol. Sci..

[9] Bindels L.B., Delzenne N.M., Cani P.D., Walter J. (2015). Towards a more comprehensive concept for prebiotics. Nat. Rev. Gastroenterol. Hepatol..

[10] Slattery M.L., Curtin K.P., Edwards S.L., Schaffer D.M. (2004). Plant foods, fiber, and rectal cancer. Am. J. Clin. Nutr..

[11] Liberati A., Altman D.G., Tetzlaff J., Mulrow C., Gotzsche P.C., Ioannidis J.P., Clarke M., Devereaux P.J., Kleijnen J., Moher D. (2009). The PRISMA statement for reporting systematic reviews and meta-analyses of studies that evaluate health care interventions: Explanation and elaboration. PLoS Med..

[12] Brown P., Brunnhuber K., Chalkidou K., Chalmers I., Clarke M., Fenton M., Forbes C., Glanville J., Hicks N.J., Moody J. (2006). How to formulate research recommendations. BMJ.

[13] Higgins J.P.T., Green S. (2013). Cochrane Handbook for Systematic Reviews of Interventions. Version 5.1.0..

[14] Egger M., Davey Smith G., Schneider M., Minder C. (1997). Bias in meta-analysis detected by a simple, graphical test. BMJ.

[15] Duval S., Tweedie R. (2000). A nonparametric “Trim and Fill” method of accounting for publication bias in meta-analysis. J. Am. Stat. Assoc..

[16] Leimu R., Koricheva J. (2004). Cumulative meta-analysis: A new tool for detection of temporal trends and publication bias in ecology. Proc. Biol. Sci..

[17] Park S.Y., Wilkens L.R., Kolonel L.N., Henderson B.E., Le Marchand L. (2016). Inverse associations of dietary fiber and menopausal hormone therapy with colorectal cancer risk in the multiethnic cohort study. Int. J. Cancer.

[18] Otani T., Iwasaki M., Hanaoka T., Kobayashi M., Ishihara J., Natsukawa S., Shaura K., Koizumi Y., Kasuga Y., Yoshimura K. (2005). Folate, vitamin B6, vitamin B12, and vitamin B2 intake, genetic polymorphisms of related enzymes, and risk of colorectal cancer in a hospital-based case-control study in Japan. Nutr. Cancer.

[19] Hansen L., Skeie G., Landberg R., Lund E., Palmqvist R., Johansson I., Dragsted L.O., Egeberg R., Johnsen N.F., Christensen J. (2012). Intake of dietary fiber, especially from cereal foods, is associated with lower incidence of colon cancer in the HELGA cohort. Int. J. Cancer.

[20] Dahm C.C., Keogh R.H., Spencer E.A., Greenwood D.C., Key T.J., Fentiman I.S., Shipley M.J., Brunner E.J., Cade J.E., Burley V.J. (2010). Dietary fiber and colorectal cancer risk: A nested case-control study using food diaries. J. Nat. Cancer Inst..

[21] Arbman G., Axelson O., Ericsson-Begodzki A.B., Fredriksson M., Nilsson E., Sjodahl R. (1992). Cereal fiber, calcium, and colorectal cancer. Cancer.

[22] Terry P., Giovannucci E., Michels K.B., Bergkvist L., Hansen H., Holmberg L., Wolk A. (2001). Fruit, vegetables, dietary fiber, and risk of colorectal cancer. J. Nat. Cancer Inst..

[23] Vulcan A., Brandstedt J., Manjer J., Jirstrom K., Ohlsson B., Ericson U. (2015). Fibre intake and incident colorectal cancer depending on fibre source, sex, tumour location and tumour, node, metastasis stage. Br. J. Nutr..

[24] Bingham S.A., Day N.E., Luben R., Ferrari P., Slimani N., Norat T., Clavel-Chapelon F., Kesse E., Nieters A., Boeing H. (2003). Dietary fibre in food and protection against colorectal cancer in the European Prospective Investigation into Cancer and Nutrition (EPIC): An observational study. Lancet.

[25] Bingham S.A., Norat T., Moskal A., Ferrari P., Slimani N., Clavel-Chapelon F., Kesse E., Nieters A., Boeing H., Tjonneland A. (2005). Is the association with fiber from foods in colorectal cancer confounded by folate intake?. Cancer Epidemiol. Biomark. Prev..

[26] Murphy N., Norat T., Ferrari P., Jenab M., Bueno-de-Mesquita B., Skeie G., Dahm C.C., Overvad K., Olsen A., Tjonneland A. (2012). Dietary fibre intake and risks of cancers of the colon and rectum in the European prospective investigation into cancer and nutrition (EPIC). PLoS ONE.

[27] Fuchs C.S., Giovannucci E.L., Colditz G.A., Hunter D.J., Stampfer M.J., Rosner B., Speizer F.E., Willett W.C. (1999). Dietary fiber and the risk of colorectal cancer and adenoma in women. N. Engl. J. Med..

[28] Schatzkin A., Mouw T., Park Y., Subar A.F., Kipnis V., Hollenbeck A., Leitzmann M.F., Thompson F.E. (2007). Dietary fiber and whole-grain consumption in relation to colorectal cancer in the NIH-AARP Diet and Health Study. Am. J. Clin. Nutr..

[29] Uchida K., Kono S., Yin G., Toyomura K., Nagano J., Mizoue T., Mibu R., Tanaka M., Kakeji Y., Maehara Y. (2010). Dietary fiber, source foods and colorectal cancer risk: The fukuoka colorectal cancer study. Scand. J. Gastroenterol..

[30] Wakai K., Date C., Fukui M., Tamakoshi K., Watanabe Y., Hayakawa N., Kojima M., Kawado M., Suzuki K., Hashimoto S. (2007). Dietary fiber and risk of colorectal cancer in the Japan collaborative cohort study. Cancer Epidemiol. Biomark. Prev..

[31] Wakai K., Hirose K., Matsuo K., Ito H., Kuriki K., Suzuki T., Kato T., Hirai T., Kanemitsu Y., Tajima K. (2006). Dietary risk factors for colon and rectal cancers: A comparative case-control study. J. Epidemiol..

[32] Shin A., Li H., Shu X.O., Yang G., Gao Y.T., Zheng W. (2006). Dietary intake of calcium, fiber and other micronutrients in relation to colorectal cancer risk: Results from the Shanghai women’s health study. Int. J. Cancer.

[33] Zhong X., Fang Y.J., Pan Z.Z., Lu M.S., Zheng M.C., Chen Y.M., Zhang C.X. (2014). Dietary fiber and fiber fraction intakes and colorectal cancer risk in Chinese adults. Nutr. Cancer.

[34] Song J.W., Chung K.C. (2010). Observational studies: Cohort and case-control studies. Plast. Reconstr. Surg..

[35] Tuncil Y.E., Nakatsu C.H., Kazem A.E., Arioglu-Tuncil S., Reuhs B., Martens E.C., Hamaker B.R. (2017). Delayed utilization of some fast-fermenting soluble dietary fibers by human gut microbiota when presented in a mixture. J. Funct. Foods.

[36] Chen T., Long W., Zhang C., Liu S., Zhao L., Hamaker B.R. (2017). Fiber-utilizing capacity varies in *Prevotella*-versus *Bacteroides*-dominated gut microbiota. Sci. Rep..

[37] Prado S.B.R.d., Castro-Alves V.C., Ferreira G.F., Fabi J.P. (2019). Ingestion of non-digestible carbohydrates from plant-source foods and decreased risk of colorectal cancer: A review on the biological effects and the mechanisms of action. Front. Nutr..

[38] Lim C.C., Ferguson L.R., Tannock G.W. (2005). Dietary fibres as “prebiotics”: Implications for colorectal cancer. Mol. Nutr. Food Res..

[39] Duvallet C., Gibbons S.M., Gurry T., Irizarry R.A., Alm E.J. (2017). Meta-analysis of gut microbiome studies identifies disease-specific and shared responses. Nat. Commun..

[40] Ahn J., Sinha R., Pei Z., Dominianni C., Wu J., Shi J., Goedert J.J., Hayes R.B., Yang L. (2013). Human gut microbiome and risk for colorectal cancer. J. Nat. Cancer Inst..

[41] Youssef O., Lahti L., Kokkola A., Karla T., Tikkanen M., Ehsan H., Carpelan-Holmstrom M., Koskensalo S., Bohling T., Rautelin H. (2018). stool microbiota composition differs in patients with stomach, colon, and rectal neoplasms. Dig. Dis. Sci..

[42] Flemer B., Lynch D.B., Brown J.M., Jeffery I.B., Ryan F.J., Claesson M.J., O’Riordain M., Shanahan F., O’Toole P.W. (2017). Tumour-associated and non-tumour-associated microbiota in colorectal cancer. Gut.

[43] O’Callaghan A., van Sinderen D. (2016). Bifidobacteria and their role as members of the human gut microbiota. Front. Microbiol..

[44] Cianci R., Franza L., Schinzari G., Rossi E., Ianiro G., Tortora G., Gasbarrini A., Gambassi G., Cammarota G. (2019). The interplay between immunity and microbiota at intestinal immunological niche: The case of cancer. Int. J. Mol. Sci..

[45] Federici E., Prete R., Lazzi C., Pellegrini N., Moretti M., Corsetti A., Cenci G. (2017). Bacterial composition, genotoxicity, and cytotoxicity of fecal samples from individuals consuming omnivorous or vegetarian diets. Front. Microbiol..

[46] Yu G., Bei J., Zhao J., Li Q., Cheng C. (2018). Modification of carrot (*Daucus carota* Linn. var. *Sativa* Hoffm.) pomace insoluble dietary fiber with complex enzyme method, ultrafine comminution, and high hydrostatic pressure. Food Chem..

[47] Wang Y., Sun P., Li H., Adhikari B.P., Li D. (2018). Rheological behavior of tomato fiber suspensions produced by high shear and high pressure homogenization and their application in tomato products. Int. J. Anal. Chem..

