# Supplementary material for: Rectal Cancer: 20% Risk Reduction Thanks to Dietary Fibre Intake. Systematic Review and Meta-Analysis
Source: Nutrients. 2019 Jul 12;11(7):1579. doi: 10.3390/nu11071579 (PMC6683071; doi:10.3390/nu11071579)
Supplement: Supplementary file 1 [file nutrients-11-01579-s001.zip › Figure S1b.pdf]

	ES	95% CI	Sig.	N
Arbman_1992	0.12	0.02 , 0.76	0.055	28
Bingham_2005	0.39	0.09 , 1.66	0.204	631
Dahm_2010	0.63	0.39 , 1.00	0.051	1495
Zhong_2014	0.53	0.34 , 0.81	0.003	2373
Slattery(b)_2004	0.53	0.39 , 0.73	0.000	3318
Slattery(a)_2004	0.54	0.42 , 0.68	0.000	4550
Uchida_2010	0.61	0.44 , 0.84	0.003	6181
Wakai(a)_2006	0.63	0.48 , 0.82	0.001	8958
Vulcan(a)_2015	0.65	0.51 , 0.84	0.001	36889
Hansen(a)_2012	0.70	0.54 , 0.90	0.005	75741
Wakai(a)_2007	0.72	0.57 , 0.92	0.009	118856
Otani(a)_2005	0.73	0.58 , 0.92	0.008	166256
Otani(b)_2005	0.74	0.59 , 0.92	0.008	219794
Terry_2001	0.77	0.62 , 0.95	0.014	281257
Hansen(b)_2012	0.78	0.64 , 0.95	0.015	350486
Shin_2006	0.79	0.65 , 0.95	0.013	425428
Fuchs_1999	0.78	0.65 , 0.93	0.006	514185
Park(b)_2016	0.76	0.64 , 0.90	0.001	615954
Bingham_2003	0.76	0.65 , 0.89	0.001	1050163
Murphy_2012	0.77	0.67 , 0.89	0.000	1527475
Schatzkin_2007	0.80	0.69 , 0.92	0.002	2017086
Park (a)_2016	0.77	0.66 , 0.89	0.001	2876136

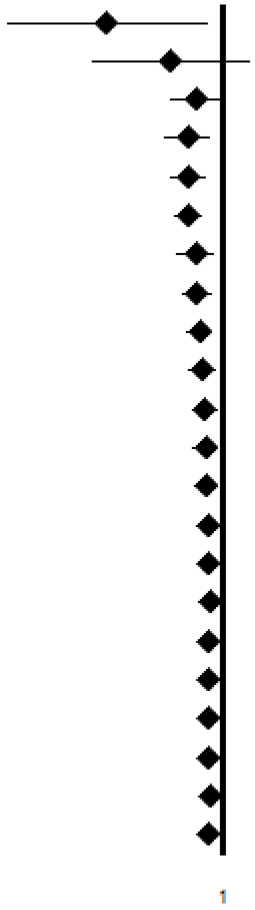

Figure S1b Forest plot of the cumulative analysis for sample size in prevention of rectal cancer
