# Supplementary material for: Rectal Cancer: 20% Risk Reduction Thanks to Dietary Fibre Intake. Systematic Review and Meta-Analysis
Source: Nutrients. 2019 Jul 12;11(7):1579. doi: 10.3390/nu11071579 (PMC6683071; doi:10.3390/nu11071579)
Supplement: Supplementary file 1 [file nutrients-11-01579-s001.zip › Figure S2.pdf]

	ES	95% CI	N
Arbman_1992	0.12	0.02 , 0.76	28
Bingham_2003	0.80	0.53 , 1.22	434209
Fuchs_1999	0.63	0.37 , 1.08	88757
Slattery(a)_2004	0.54	0.34 , 0.86	1232
Slattery(b)_2004	0.54	0.29 , 0.98	945
Terry_2001	1.10	0.69 , 1.76	61463
1990-2004	0.69	0.51 , 0.92	586634
Bingham_2005	0.62	0.44 , 0.87	603
Dahm_2010	0.82	0.40 , 1.66	864
Otani(a)_2005	0.95	0.40 , 2.30	47400
Otani(b)_2005	1.00	0.32 , 3.30	53538
Schatzkin_2007	1.13	0.84 , 1.51	489611
Shin_2006	0.90	0.40 , 2.10	74942
Uchida_2010	1.24	0.74 , 2.10	1631
Wakai(a)_2006	0.72	0.49 , 1.08	2777
Wakai(a)_2007	1.10	0.59 , 2.07	43115
2005-2010	0.89	0.74 , 1.08	714481
Hansen(a)_2012	1.11	0.74 , 1.66	38852
Hansen(b)_2012	0.97	0.66 , 1.42	69229
Murphy_2012	0.90	0.72 , 1.14	477312
Park (a)_2016	0.47	0.35 , 0.62	859050
Park(b)_2016	0.62	0.45 , 0.86	101769
Vulcan(a)_2015	0.88	0.55 , 1.42	27931
Zhong_2014	0.38	0.24 , 0.60	878
2011-2015	0.71	0.54 , 0.95	1575021

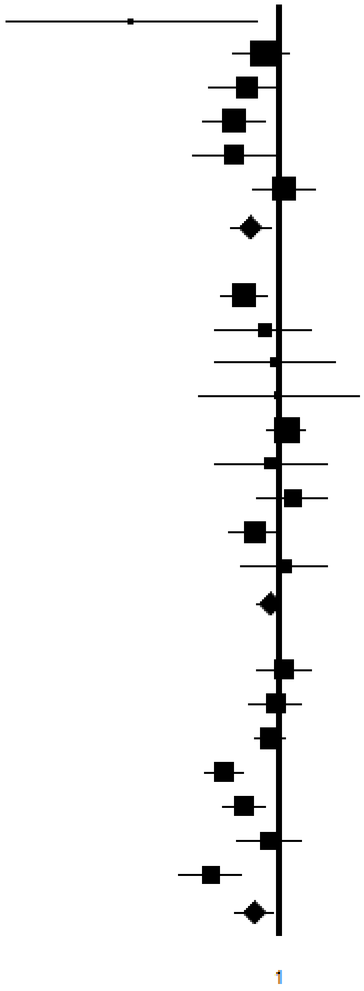

Figure S2 Forest plot of the moderator analysis for year of publication in prevention of rectal cancer
