# Supplementary material for: Rectal Cancer: 20% Risk Reduction Thanks to Dietary Fibre Intake. Systematic Review and Meta-Analysis
Source: Nutrients. 2019 Jul 12;11(7):1579. doi: 10.3390/nu11071579 (PMC6683071; doi:10.3390/nu11071579)
Supplement: Supplementary file 1 [file nutrients-11-01579-s001.zip › Table S1.docx]

**Table S1. Detailed inclusion/exclusion criteria, according to PICOS statement extended with language and time filter**.

| Search strategy | Details |
| --- | --- |
| Inclusion criteria | P: adult population (female and male)  I: questionnaire/interview measuring dietary fiber intake  C: the highest vs the lowest intake  O: risk of rectal cancer (if any)  S: primary studies (clinical trial, cohort, case-control, cross-sectional) |
| Exclusion criteria | P: infants, children and adolescents, colon-rectal cancer combined or colon cancer alone.  I: no assessment of dietary intake  O: other outcomes not related to rectal cancer risk  S: not original papers (opinion paper, review article, commentary, letter, article without quantitative data) |
| Language filter | English |
| Time filter | No filter (from inception) |
| Database | PubMed/Medline; EMBASE; Scopus |
