# Supplementary material for: Rectal Cancer: 20% Risk Reduction Thanks to Dietary Fibre Intake. Systematic Review and Meta-Analysis
Source: Nutrients. 2019 Jul 12;11(7):1579. doi: 10.3390/nu11071579 (PMC6683071; doi:10.3390/nu11071579)
Supplement: Supplementary file 1 [file nutrients-11-01579-s001.zip › Table S2.docx]

**Table S2. Quality evaluation of included studies (Low, Unclear, High) according to Higgins, J. P.T. , and S. Green. *Cochrane Handbook for Systematic Reviews of Interventions. Version 5.1.0.*: The Cochrane Collaboration, 2013**

| **Author year** | **Selection bias** | **Performance bias** | **Detection bias** | **Attrition bias** | **Reporting bias** |
| --- | --- | --- | --- | --- | --- |
| Arbman 1992 | High | Low | Low | Low | Low |
| Bingham 2003 | Low | High | Low | Low | Low |
| Bingham 2005 | Low | High | Low | Low | Low |
| Dahm 2010 | Low | Low | Low | High | Low |
| Fuchs 1999 | Low | Unclear | Low | Low | Low |
| Hansen 2012 | Low | Unclear | Low | Low | Low |
| Murphy 2013 | Low | Unclear | Low | Low | Low |
| Otani 2005 | Low | Unclear | Low | Unclear | Low |
| Park 2016 | Low | Low | Low | High | Low |
| Schatzkin 2007 | Low | Unclear | Low | Low | Low |
| Shin 2006 | Low | Unclear | Low | Low | Low |
| Slattery 2004 | Low | Unclear | Low | Low | Low |
| Terry 2001 | Low | Unclear | Low | Low | Low |
| Uchida 2010 | Low | Unclear | Low | Low | Low |
| Vulcan 2015 | Low | Low | Low | Low | Low |
| Wakai 2006 | Low | Unclear | Low | Low | Low |
| Wakai 2007 | Low | Unclear | Low | Low | Low |
| Zhong 2014 | Low | Unclear | Low | Low | Low |
