# Supplementary figures and images for: Rectal Cancer: 20% Risk Reduction Thanks to Dietary Fibre Intake. Systematic Review and Meta-Analysis
Source: Nutrients. 2019 Jul 12;11(7):1579. doi: 10.3390/nu11071579 (PMC6683071; doi:10.3390/nu11071579)

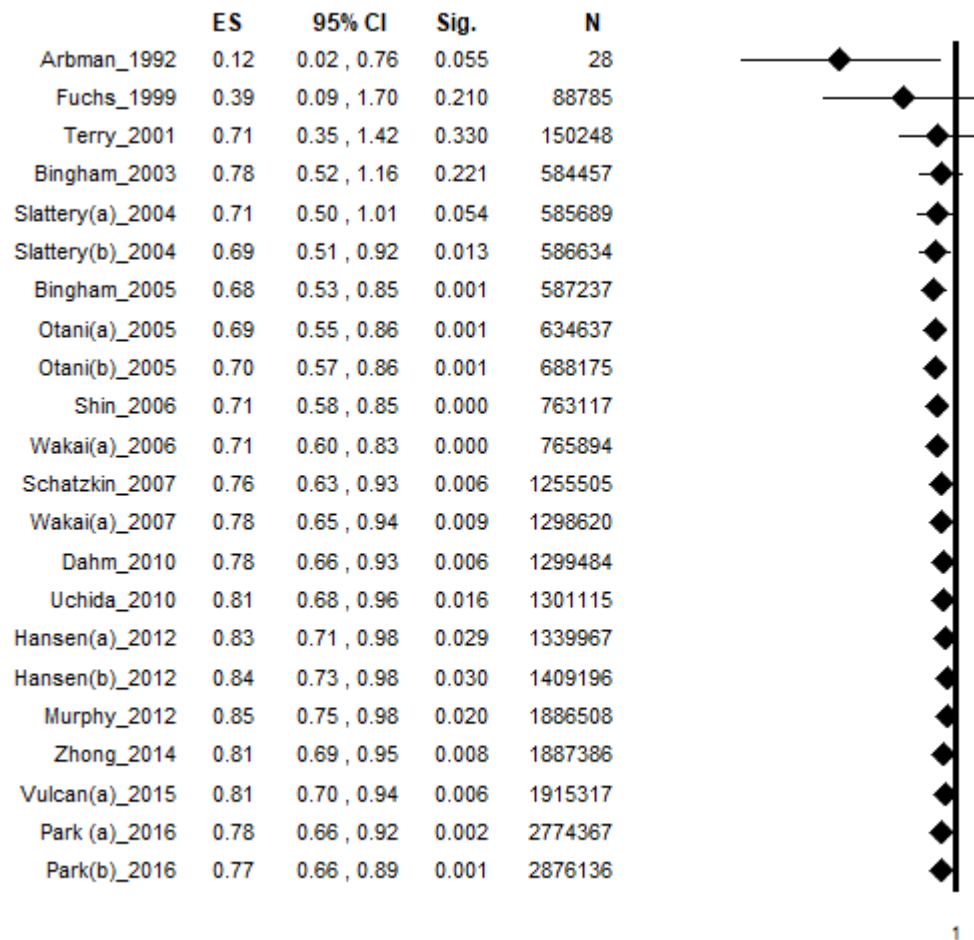

Figure S1a Forest plot of the cumulative analysis for year of publication

Supplement: Supplementary file 1 [file nutrients-11-01579-s001.zip › Figure S1a.pdf]
